# Report of 106 Cases of Pneumonia at the Naval Hospital, League Island, Pa.

**Published:** 1919

**Authors:** 


***Report of 106 Cases of Pneumonia at the Naval Hospital, League Island, Pa.*** By Lieut. F. J. DEYER and Lieut. M. C. BOLES. *U. S. Naval Medical Bulletin*, October 1918.

The pneumonia was of the usual clinical variety, in no case secondary to measles. The total number of cases was 106, of which 90 were of the lobar variety, and 16 of the bronchial variety. Total deaths from all causes 27, mortality 25.4 per cent.; deaths actually attributed to lobar pneumonia, 15, mortality rate 14.1 per 100.

Determination of the type of the causative organism made in 68 cases: 11 cases, Type 1; 4 cases, Type 2; 9 cases, Type 3; 39 cases, Type 4; 5 cases, streptococcus. Type 3 was the most virulent in these cases; Type 4 next in virulence.

In 80 cases the diseases commenced with a chill; in 26 cases, onset was gradual.

Empyema occurred in 25 per cent. of cases, all following lobar pneumonia. Other complications were numerous and severe. Otitis media was not as frequent as in camps where streptococcus infection prevailed.

The mortality rate from empyema was low, chiefly because the pneumococcus, not the streptococcus infections, prevailed; also on account of prompt treatment. Exploratory incision was made in all cases of pneumonia in which the crisis did not occur by the eleventh day. Upon the discovery of turbid fluid in the pleural cavity, a rib resection or thoracotomy was performed. The authors believe in thorough surgical drainage in empyema when turbid fluid is found, instead of repeated aspirations until thick pus develops.

Treatment was largely symptomatic; little medication was used. Stimulation was not used unless indicated by the character of the second pulmonic sound, and the quality of the muscular element of the first sound of the heart. When necessary, strychnine was used hypodermatically. Open air treatment is recommended in lobar pneumonia but not in the bronchial type. Vaccines are not to be used, but the use of specific serum in Group 1 pneumonias is strongly advised. Initial dose used is 50 to 150 cc. depending on severity of the infection.

In diet, the appetite of the patient was the guide. Any patient who had a desire for food was given soft-boiled or poached eggs, toast, rice, junket, etc., regardless of degree of fever. Those who had no inclination for food were fed on broths and milk.

